# Ajyal Salima a novel public–private partnership model for childhood obesity prevention in the Arab countries

**DOI:** 10.3389/fpubh.2022.1012752

**Published:** 2022-12-06

**Authors:** Carla Habib-Mourad, Nahla Hwalla, Carla Maliha, Sarah Zahr, Karine Antoniades

**Affiliations:** ^1^Department of Nutrition and Food Sciences, Faculty of Agriculture and Food Sciences, American University of Beirut, Beirut, Lebanon; ^2^Nestlé Middle East FZE, Dubai, United Arab Emirates

**Keywords:** childhood, nutrition, obesity, school-based intervention, health promotion, Arab region, partnership

## Abstract

The prevalence of childhood overweight and obesity among children is on the rise around the world. Meanwhile, comprehensive multi-sectorial approaches have been found to be effective in improving nutritional status among children. Ajyal Salima is a public–private partnership (PPP) school-based nutrition and physical activity intervention program implemented in six Arab countries. Its objective is to promote healthy eating and physical activity habits among 9–11-year-old students. The stakeholders, involved with the implementation of the program, comprised (1) local authorities, ministries of Education and Health, and non-governmental organizations (NGOs) as public partners, (2) The American University of Beirut (AUB) as the academic/regional scientific partner, and (3) Nestlé as the private partner. The Ajyal Salima program encompasses four coordinated educational components: classroom sessions, family involvement, food service intervention, and training of trainers. The program's educational material has been culturally adapted to each country's needs, as well as pilot tested. This paper describes the strategies used to build the PPP framework of Ajyal Salima, and the role of each stakeholder. The Ajyal Salima program is an example of a promising and sustainable comprehensive PPP program to address childhood obesity, that can be exported to other countries in the region and globally.

## Introduction

The world is facing a progressive rise in the global prevalence of obesity, particularly childhood obesity that has increased from 31 to 42 million as reported by the World Health organization (WHO) (1). In the Arab region, a triple burden of malnutrition exists including undernutrition, micronutrient deficiency, and over nutrition. This region has elevated rates of obesity and non-communicable diseases (NCDs) that are associated with the rapid economic, social, and political changes in related countries. Socio-economical changes have possibly led to a nutrition transition and transformation in the lifestyle of people in the region (2). Urbanization, technological development, and modernization have reportedly led to shifts in dietary habits, physical activity, and increased NCDs, particularly among children in the region (2). A striking increase in the rates of overweight and obesity have been noted in children in the Arab region where 25–40% of children and adolescents were reported to be overweight or obese (1, 3). Holistic interventions were reported as needed to modify the obesogenic food environment and facilitate adequate food choices for this vulnerable population (4). Targeting childhood overweight and obesity has become essential to help resolve public health problems, requiring urgent evidence-based approaches to reverse the trend (1). Programs designed to address the obesogenic environment need to be tailored to take into consideration cultural peculiarities and relevant food environments to achieve progress in mitigating childhood obesity.

According to the United Nations, a multisectoral approach that integrates various stakeholders, such as governments, local policy makers, health sectors, and civil society, is recommended to address malnutrition in children (5). This approach, known as the public–private partnerships (PPP), is defined as mobilization of funds from the private sector to governmental or non-governmental organizations (NGOs) to enhance their generally declining spending on public health issues (6). To be deemed successful, PPPs targeting childhood obesity need to be culture specific, encourage local engagement and long-term commitment, and include multiple stakeholders. Additionally, PPPs need to be evidence-based, undergo continuous monitoring and evaluation, and ensure that all stakeholders have access to program information and reports (7). Many reports from other countries have proven the PPP to be an effective tool in tackling the double burden of childhood obesity, specifically when a partnership is school based (8). Internationally, several PPP protocols for preventing childhood obesity that address dietary and sedentary behaviors were implemented in the United States (9–12), the United Kingdom (13), and Europe (14, 15). As for the Arab region, intervention programs targeting childhood overweight and obesity mostly focus on dietary modifications, while overlooking the interplay between behavioral, environmental, and psychological factors (16).

Ajyal Salima, which translates in English to “Healthier Kids,” constitutes the region's sole PPP-based, multi-component, and holistic school-based nutrition intervention program to address the obesogenic environment. This paper describes the strategies and methodologies used to build the program's PPP framework, and the role of each stakeholder. It also serves as a model for the future development of similarly effective private–public partnerships to tackle rising obesity rates among schoolchildren elsewhere.

## Methodology

### Establishing partnerships and process of implementation

The Healthier Kids-Ajyal Salima school program was initially developed in 2008 by the American University of Beirut (AUB) as “Health-E-PALS” to tackle childhood obesity in Lebanon through addressing nutritional and physical activity habits of schoolchildren (17). The first Ajyal Salima PPP was established in Lebanon in 2010 between Nestlé Middle East FZE and AUB to expand the program's national coverage. The Lebanese Ministry of Education and Higher Education then joined as the public partner, helping Ajyal Salima further develop into a national program with its formal adoption by the Ministry in public schools. The research team at AUB was responsible for teacher training, monitoring and evaluation of the program implementation in both public and private schools.

Following the pilot year, and with the scientific support of AUB, Nestlé set out to expand the program's implantation in the region, engaging with different stakeholders and authorities, disseminating learnings from the program, putting together proposals for PPP collaborations, as well as providing the evidence of its effectiveness in promoting nutrition knowledge and enhancing healthy eating in school children (18). The PPP model and evidence-based results from the pilot study constituted the foundation to help establish collaboration and partnerships between local authorities and Nestlé in the United Arab Emirates (UAE), Kingdom of Saudi Arabia (KSA), Jordan, Palestine, and Bahrain. Individual agreements and frameworks on action plans tailored the program's governance and elements to each's specific needs, providing the set-up for local implementation, roles and responsibilities, monitoring, and evaluation. To roll out the program in these Arab countries, and in light of specific cultural peculiarities and local needs of the countries involved, the original program components were modified to suit local traditions, different types of food consumed, and their local names.

In the UAE, Palestine, and Bahrain, agreements were signed between governmental partners (Ministry of Health, Ministry of Education, and the Dubai Health Authority) and the private partner Nestlé Middle East FZE. The program was integrated into Palestinian strategic education and school health action plans. In Jordan and Saudi Arabia, the agreements were signed between Nestlé Middle East FZE and NGOs and agencies, the Royal Health Awareness Society (RHAS) in Jordan and Tatweer Education Holding in Saudi Arabia. The ministries in both Jordan and Saudi Arabia were also included in this collaboration framework. Table 1 presents the timeline for the Ajyal Salima rollout in various locations as well as respective partners and relevant stakeholders in each.

**Table 1:** Timetable for the Healthier Kids-Ajyal Salima rollout in the selected Arab countries with the relevant stakeholders involved.

**Year**	**Program roll-out**
2010	Launch of the program in Lebanon Partners: American University of Beirut Ministry of Education and Higher Education Nestlé Middle East FZE
2012	Pilot study of the program in UAE Partners: American University of Beirut Dubai Health Authority Princess Haya initiative Dubai Education Zone Nestlé Middle East FZE
2014	Pilot study of the program in KSA Partners: American University of Beirut Ministry of Education Tatweer Educational Holding Company Nestlé Middle East FZE
2014	Adoption of the program by the Lebanese Ministry of Education and Higher Education into their health education unit
2015	Launch of the program in Jordan Partners: American University of Beirut Ministry of Education Ministry of Health Non-Governmental organization: Royal Health Awareness Society (RHAS) Nestlé Middle East FZE
2016	Launch of the program in Palestine Partners: American University of Beirut Ministry of Education Nestlé Middle East FZE
2018	Launch of the program in Bahrain Partners: American University of Beirut Ministry of Health Ministry of Education Nestlé Middle East FZE

**Figure 1:**
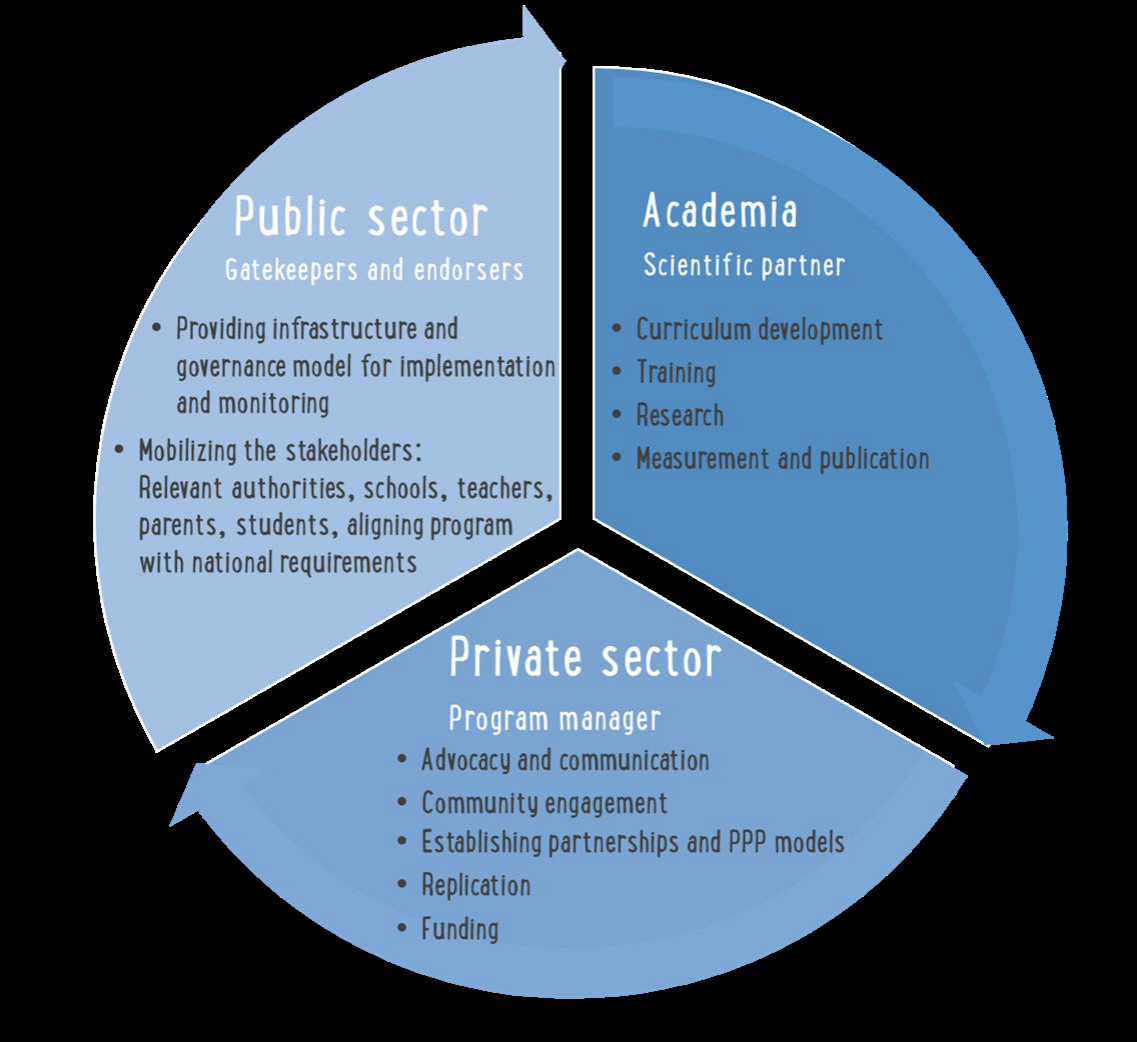
The Ajyal Salima private–public partnerships (PPP) model.

The role of each stakeholder is outlined in Figure 1, which describes the Ajyal Salima PPP. This partnership consisted of (1) Local authorities, mainly the Ministries of Education and Health, in addition to NGOs, which served as the country's public partners, endorsers, and gatekeepers. These authorities ensured that the program was embedded in relevant infrastructure, contributed to the local health strategy objectives, included in school health roadmaps, and ensured sustainability. Moreover, the engaged ministries secured access to schools and established on-site supervisors who followed up on the implementation of the Ajyal Salima program within them. (2) The AUB as the academic/regional scientific partner and coordinator provided the material for scientific dissemination, curriculum content development and adaptation, training of trainers, data analysis, and scientific publications. (3) Nestlé Middle East FZE, as the private partner, contributed to the development of the PPP model as part of the Nestlé for Healthier Kids Global Initiative and in line with its Creating Shared Value strategic approach. It led communication across the region, established partnerships, and supported logistics for replication, and expansion of the program; however, Nestlé did not play a role in the program's content, implementation and delivery nor in schools/students selection, segregation of data, or data analysis and reporting.

### Process of development of Ajyal Salima intervention program

The Ajyal Salima program is a multi-component school-based intervention that relies on the Social Cognitive Theory, which goes beyond the acquisition of knowledge to include environmental modifications that support individual behavioral changes (19). The program has four coordinated intervention components that address an individual's behavior change including knowledge, skills, self-efficacy, environmental factors such as reinforcement of good behavior, modeling of significant others, as well as availability of recommended foods at home and in the school environment. These components were structured to work together to address behavioral and environmental factors related to students' dietary and physical activity behaviors in the school and at home. Consistent with the Social Cognitive Theory, the components were based on the expectation that children will make healthier choices when introduced in a social setting that includes family and peers as well as using active learning strategies.

### Intervention components

The Ajyal Salima intervention targets encouraging consumption of nutrient rich foods and specific energy balance-related behaviors such as physical activity, sedentary behaviors, and dietary intake that play a significant role in energy balance leading to weight gain in 9–11-year-old children (20, 21). Specifically, the intervention focused on addressing: (1) Increasing consumption of fruits and vegetables (2), Favoring healthy snacks over high energy dense snacks and drinks (3), Consuming a healthy breakfast daily (4), Increasing moderate physical activity (5), Decreasing sedentary behavior.

The four intervention components included:

1. Interactive classroom sessions: Culturally appropriate educational lessons, included fun and attractive material, designed to promote healthy eating, and physical activity, delivered by trained teachers. This component covered the personal and psychosocial determinants as outlined by the Social Cognitive Theory (19).
2. Parental involvement: The intervention program was introduced to families to assist them in creating a supportive environment at home for healthy lifestyle behaviors. Take-home packets including nutrition and physical activity tips, as well as recipes were sent with the students. Additionally, parents were invited to health fairs organized at the school. This component covered the obesogenic environment at home.
3. Food service intervention: This component covered the availability of food in the school environment and provided relevant recommendations to include a healthy list of snacks and drinks and exclude unhealthy choices for children in the school shop.
4. Train the trainers (TTT) workshops: workshops were conducted in each country and consisted of a 2-day interactive face-to-face training of teachers, by a research team of dietitians, on all program components and hands-on coaching and role-plays on all educational activities. Teachers and health educators in each country were coached on the use of a complete tool kit, which consisted of a “Teacher's guide” with lesson plans and educational material (posters, pamphlets, booklets…) to ensure the delivery of the intervention exactly as designed. In addition, school-specific implementation plans were discussed and agreed upon with teachers and school administration along with hands-on exercises, illustrative of the program. Table 2 presents the intervention components, the number of sessions or meetings per year for each component and the target population.

**Table 2:** Intervention components and target population.

**Program's components**	**Number of sessions/meetings per year**	**Target population**
Classroom sessions	**12 modules over the year:** 15–20 min lesson 20–25 min activity	Students (9–11-year-old)
Family program	**2–3 interventions included:** Parents meeting to introduce the programme Parents participation in an in-class activity Invitation for parents over breakfast End-of-year school event	Parents
School canteens	**Intervention included:** Providing healthy food choices Offering less energy dense snacks and drinks options	Food service personnel
Train the trainers	**2-day workshop:** Training provided by AUB research team Material included: Lesson plans, activity sheets, support material for parental meetings, and schools shops	Teachers and health educators

### Program monitoring and evaluation

The outlined PPP model was designed to be implemented across all six countries, with results used to further expand it to other countries. Collectively, the Ajyal Salima PPP brought together ten partners across six countries and reached 300,000 schoolchildren, their parents, and teachers. Given that this was a large-scale PPP, it was vital to monitor the program and evaluate its efficacy to expand it into other countries. The evaluation process included a pre-test 1 week prior to the start of the intervention and a post-test 1 week after the completion of the program (3–4 months duration) in all schools. These tools were developed by AUB as the scientific partner, which is the custodian of the data that will be later analyzed and shared with all partners. Evaluation and monitoring involved all partners and stakeholders to ensure program sustainability and success. Ethical approval of the study was granted by AUB's Institutional Review Board in Lebanon. Additional approvals were obtained by the Ministries of Health and Education in Jordan, the Palestine, Bahrain, and Saudi Arabia.

## Discussion and conclusion

This paper describes the Ajyal Salima regional program as a process and a model for implementing a PPP addressing childhood obesity and promoting healthy eating and physical activity in schoolchildren in different administrative settings. The Ajyal Salima program, which relied on the Social Cognitive Theory, is one of the first multi-component school-based interventions to be implemented in the Arab region that highlighted the importance of PPPs in addressing prevention of childhood obesity. It has been conceived as a gradual process involving development, piloting, adoption, rollout, and expansion through partnerships between different stakeholders from several Arab countries.

Public–private partnerships, now commonly used in the health sector, have been shown to be effective in promoting sustainable health programs, by enriching the capacity, quality, and reach of public health services and allowing innovation in the dissemination of health-related messages (22).

In the Ajyal Salima model, an integral part of the PPP was the research team at AUB as the academic partner responsible for all scientific aspects of the program, such as developing educational material and project tools, training of trainers, and monitoring and evaluation. A similar PPP is the Ensemble Prévenons l'Obésité Des Enfants (EPODE); a community-based program launched in France in 1992 involving an academic partner, as well as a public partner and NGOs (23). Four universities and numerous specialists/academics from different European countries were included in the partnership. The central coordination team, composed of specialists, coordinated the program, developed all the tools, and conducted continuous monitoring and evaluation (15). According to the EPODE experience, the PPP was a major factor for the success of the community-based methodology (23). Another example is the Food Hero model, where the research partner was responsible for drafting all survey tools, recruiting participants, conducting focus groups, analyzing data, and publishing progress reports (12). Additionally, the United for Healthier Kids (U4HK) program mobilized social media and social marketing to tackle childhood obesity in 11 countries (7). The U4HK program extensively relied on academic partners to develop science-based behavioral goals and develop the overall framework (7). In all PPPs scientific partners mobilize resources and share experiences to ensure the program's success. They are also responsible for the dissemination of results through research publication. The scientific evaluation is an integral part of a PPP as it ensures program's sustainability and encourages political involvement (23).

In all six countries that were included in the Ajyal Salima PPP model, there was a local public partner endorsing the program, mainly the local ministry of education or the ministry of health, or both simultaneously. Involving a public partner is important to ensure that the program is culture sensitive and abides by local rules, regulations, and local policies. Public partners have the authority and ability to mobilize large scale networks to secure implementation and sustainability of the program. For example, in the present PPP model, public partners secured schools' participation in the program and, in Lebanon and Palestine, embedded the model into their school curriculum. Similarly, the InFANT Program in Australia was adopted and endorsed by eight local governmental areas (24). Likewise, the U4HK program had different public partners in each participating country. One example is Mexico, where the public partners were the Ministries of Public Education and Health. In the Philippines, the Food and Nutrition Research Institute, the Department of Tourism, and the Central Bank were public partners (7). In other cases, public partners funded the intervention such as with the Pro Children program where governmental organizations were responsible for the recruitment of schools and funded the program across nine European countries (14). Public partners have a central role in any PPP addressing childhood obesity because they are the ultimate custodian of health in the country and they set policies, manage public schools, and have the leverage to make childhood obesity prevention a priority (23).

Another indispensable component of a PPP is the private sector, from which partners engage with stakeholders, create partnerships and program set-up for replication, mobilize resources, funds, and expertise to serve the program. The private partner in the Ajyal Salima model, Nestlé Middle East FZE, initiated and funded the program, and connected all involved partners from the region to achieve a common goal. Program communication was the responsibility of the private partner, which was also the case in the Change4Life PPP in which marketing agencies were recruited to create a social movement (13). Similarly, in the Food Hero PPP, the private marketing partner managed the program and employed its expertise to assist in creating it (12). Alternatively, other programs were solely dependent on the private partner, such as the Fuel up to Play program where two private partners, in collaboration with experts and school stakeholders, were responsible for program coordination and social marketing (10). Research has shown that there is a crucial need to involve the private sector in interventions targeting childhood obesity since this sector creates changes in the food supply and food environment and can lead to a positive evolution in product reformulation and advertising aimed at children (7). Many PPPs aiming to combat hunger and increase food security have been funded by large food companies because of their wide reach as they operate globally and can implement large scale programs (25).

Many PPPs started up as small-scale programs, such as the Food Hero and the Fuel Up to Play, later expanding to reach a wider audience (10, 12). The EPODE program was piloted in 10 French communities, and now operates in over 500 communities across the globe (15). Likewise, Ajyal Salima began as a pilot study in Lebanon in 2010 and now, over a decade later, continues to run in four countries. The key to a successful PPP is including both public and private sector partners along with NGOs and scientific experts from civil society. Open communication channels and well-structured protocols are also needed to ensure that all partners are working together through shared objectives, and by compelling/pooling resources and sharing the risks as well as benefits. Either public or private partners can lead or fund the program depending on resource availabilities and expertise. Ideally funding should be provided from the public sector, however, this is not always the case especially in low and low to middle income countries due to lack of governmental funds. Additionally, funding from private grants is usually for a longer duration which ensures better program sustainability (15). Likewise, interventions without the involvement of the public sector are not sustainable. The public partner ensures a program's sustainability through providing the necessary logistics and recruitment support. Some challenges or miscommunications might arise between the private and public partner, hence the importance of transparency, formal commitment, patience, and mutual trust. For example, in the Ajyal Salima PPP, detailed memorandums of understanding were signed across partners that included regulations, responsibilities, and rights. Partners also shared continuous progress reports and made sure that all information was available and accessible. Lastly, communication issues might arise when the program is running in various sites/countries with a different private partner in each. This was not the case in the context of Ajyal Salima program, as the private partner was the same across all sites.

There had been various school-based interventions in the region that targeted childhood overweight and obesity. For example, in Lebanon, the “Jarrib Baleha” intervention aimed to decrease intake of soft drinks and increase intake of water through interactive sessions (26). Three other school-based interventions were also implemented in Lebanon, though they targeted vulnerable populations such as Syrian and Palestinian refugees. These interventions aimed to increase nutritional knowledge among school children and provide complementary nutritious meals at school to improve school attendance rates (27–29). In Tunisia, a 3-year school-based intervention program was successful in increasing intake of fruits and vegetables and decreasing rates of overweight and obesity among children aged 11–16 (30). The Ajyal Salima PPP remains the first roadmap for addressing obesity challenges in Arab countries which allows for further emulation and program expansion into other countries across the region and possibly around the world. The model is holistic in its approach as it involved governments, schools, families, private, public, and industry sectors. The Ajyal Salima program includes multi-components, such as interactive classroom sessions, family orientation sessions, food service intervention, and training of trainer's workshops—Similar to other global interventions conducted to combat childhood obesity. All partners involved in the Ajyal Salima program were meeting their objectives. The public partners met national health strategies, specific for their country. The private partner, Nestlé Middle East FZE, was delivering on its Creating Shared Value commitments, in line with the global initiative. Similarly, the academic partner, AUB, was fulfilling its mission of providing research knowledge and introducing innovative programs to the scientific field through evidence-based research and community outreach. Additionally, the NGOs were achieving their goals of social development through inducing positive change within their communities. All partners were working systematically toward one common goal: implementing the Ajyal Salima program efficiently to ensure its highest impact and eventually decrease rates of childhood obesity. Despite its strength, this Ajyal Salima PPP model has some sustainability limitations; since the entire model relied on the engagement of three stakeholders, its effectiveness could be jeopardized in case one of the partners drops out. The active involvement and endorsement of government and local authorities' partners are critical to the program sustainability. Changes to the local educational authorities and health institutions roles and structure in the United Arab Emirates and in the Kingdom of Saudi Arabia prevented the program continuation and affected sustainability; the program continues to be implemented in four out of six countries: Lebanon, Jordan, Palestine, and Bahrain.

To ensure a wider implementation of the Ajyal Salima program, a deep evaluation of the efficacy and acceptability of the program components has been conducted across different countries and contexts, with results of the full-scale data analysis to be shared at a later stage.

In conclusion, this paper provided a detailed description for the implementation of a holistic school-based PPP intervention model addressing the food environment for the promotion and adoption of healthy eating and physical activity. Further expansion of the Ajyal Salima program may mitigate obesity and improve health of the Arab population; it could contribute to the existing body of literature espousing the possible success of multi-component intervention programs that include multiple partners and stakeholders from both the public and private sectors to prevent childhood obesity.

## Data availability statement

The original contributions presented in the study are included in the article/supplementary material, further inquiries can be directed to the corresponding author/s.

## Author contributions

CH-M and NH conceived the original idea and developed it, provided detailed feedback on the full manuscript, and contributed to revisions. CH-M wrote the original draft. All authors contributed to the article and approved the submitted version.

## Funding

This research was funded by Nestlé Middle East FZE without any further role in the scientific research design Grant number: 100119.

## Conflict of interest

This study received funding from Nestlé. The funder had the following involvement with the study: Contributed to the development of the PPP model as one of the program stakeholders. They led coordination efforts across the region to establish partnerships and supported the logistics of the program. All authors declare no other competing interests.

## Publisher's note

All claims expressed in this article are solely those of the authors and do not necessarily represent those of their affiliated organizations, or those of the publisher, the editors and the reviewers. Any product that may be evaluated in this article, or claim that may be made by its manufacturer, is not guaranteed or endorsed by the publisher.
